# Predictive association between immigration status and chronic pain in the general population: results from the SwePain cohort

**DOI:** 10.1186/s12889-020-09546-z

**Published:** 2020-09-29

**Authors:** Elena Dragioti, Konstantinos Tsamakis, Britt Larsson, Björn Gerdle

**Affiliations:** 1 Pain and Rehabilitation Centre, and Department of Health, Medicine and Caring Sciences, Linköping University, Linköping, Sweden; 2 King’s College London, Institute of Psychiatry, Psychology and Neuroscience, London, UK

**Keywords:** Immigration, Chronic pain, Widespread pain, Health status, Mediation analysis

## Abstract

**Background:**

Previous studies suggest that immigration may influence the experience of pain.

**Objective:**

This population-based study examines whether immigration status is associated with chronic pain (CP), chronic widespread pain (CWSP), and severe CP at a two-year follow-up. We also tested mediation by mood status (i.e., anxiety and depression).

**Methods:**

15, 563 participants from a representative stratified random sample of 34,000 individuals living in south-eastern Sweden completed a postal survey, during 2013–2015, that included the following data: immigration status; presence of CP (pain lasting at least 3 months) and CWSP (a modified classification of widespread pain for use in epidemiological studies); severity of CP based on a numeric rating scale; and depression, anxiety, economic situation, and sociodemographic information. We applied logistic regressions using the generalized estimating equations (GEE), with Swedish-born as the reference group and path analyses models.

**Results:**

Compared to the Swedish-born participants (*n* = 14,093;90%), the immigrants (*n* = 1470;10%) had an elevated risk of all pain outcomes (CP: odds ratio [OR] = 1.18; 95% confidence interval [CI = 1.04–1.33, CWSP: OR = 1.39; 95% CI: 1.15–1.69 and severe CP: 1.51; 95% CI: 1.23–1.87) after adjustments. Path analyses showed that baseline age, immigrant status, and financial hardship had a significant influence on chronic pain outcomes at follow-up with baseline mood status as the mediator. Immigration status was also associated with age and financial hardship.

**Conclusion:**

Immigrants may have increased risk of chronic pain, widespread pain, and severe pain and this risk is mediated by mood status. Targeted interventions better tailored to the socio-economic and psychological status of immigrants with chronic pain are warranted.

## Background

Immigration is considered to be a major determinant in health disparities [1, 2]. Several studies have demonstrated an association between immigration and mental well-being [3–7]. For example, the risk of developing psychosis is about two times higher in immigrants and about three times higher in immigrants from developing Eastern European countries and developing countries with high and middle income [6]. The prevalence of posttraumatic stress disorders and depression is also high among immigrants [3, 7]. Previous research using longitudinal data has also shown that immigrants are generally at higher risk of poor health [8] (e.g., ischaemic heart disease, diabetes, and stroke) [9–11]. Additionally, being an immigrant has been associated with a number of psychosocial issues such as economic stress, difficulties in adaptation, increased ambiguity for the future, changes in living conditions and in personal ties, and disruptions of usual social roles and networks [12–15].

Previous research has also highlighted the association between immigration and chronic health conditions. Understanding how immigration status and chronic health conditions are related is important because both immigration and chronic pain constitute highly complex topics that have an enormous impact on both individuals and society [11]. Emerging evidence has revealed that the prevalence of chronic pain is high among immigrants [16–20]. For example, Soares et al. [18] found that non-western born immigrants residing in Sweden experience a greater impact of chronic pain than their Swedish-born counterparts. A study from the UK found that south Asian ethnicity (i.e., people who define themselves as being of Indian, Pakistani, or Bangladeshi origin) was a significant predictor of spinal pain with disability [16], while other studies have shown that immigrants have higher odds of social difficulties, chronic widespread musculoskeletal pain [19, 21], higher pain-related psychological consequences, and higher rates of pain-related disability [16, 18, 19]. Kurita et al. [19] also found that immigrants in general report a higher pain prevalence and higher pain intensity than native-born individuals.

However, the causal pathway between immigration and chronic health outcomes remains not entirely clear, as the reasons that immigrant status seems to be associated with an increased risk for pain appear to be multifaceted and previous studies have shown some contradictory results. Choudhury et al. [20] found that Bangladeshi ethnic minority group in East London who have lower levels of acculturation (i.e. assimilation to a different culture, typically the dominant one) experience more pain, however other studies have shown that increased acculturation in immigrants is associated with higher reports of chronic back and neck problems [17]. In general, it has been suggested that the stress of the immigrant experience can lead to a higher report of chronic back or neck problems among immigrant respondents [17]. Given the lower use of anxiolytics and opioids in immigrants in Denmark who report increased pain, a previous study has questioned whether immigrants are undertreated, or whether healthcare professional attitudes and lack of resources are contributing to reduced access to care for immigrant populations [19]. Finally, the fact that immigrants are more likely to work in riskier jobs with poor working conditions which are more physically strenuous and demanding can be another potential explanation for the increased pain among immigrants [22].

Immigrants in Sweden have poor somatic health, including musculoskeletal disorders, compared to native-born Swedes, and are over-represented among those who get an early retirement due to musculoskeletal disorders [21]. A Swedish study investigating patient reported outcomes after hip arthroplasty highlighted that immigrant groups indicated more pain than those born in Sweden [23]. The differences between immigrants and native-born Swedes can be due to immigrant specific factors (e.g., discrimination, cultural adjustment, language), whilst a further, noticed association between poor health and being born in a country other than Sweden, was greatly reduced when the social network, social support, and economic factors were controlled for [21].

However, one line of research indicates that foreign-born status may represent a health advantage, a phenomenon known as the ‘healthy immigrant effect’ [24]. A systematic review of healthcare outcomes in Canada determined that on average the immigrant population is healthier than the Canadian-born population in terms of mental health, chronic conditions, disability/functional limitations, and risk behaviours [25]. Furthermore, as immigrants worldwide are increasing, studies are needed that examine health problems such as chronic pain among immigrants [12, 13].

To this end, this population-based study with a two-year follow-up investigates whether immigration status is associated with chronic pain. First, we tested the hypothesis that the odds ratio (OR) of having chronic pain at a two-year follow-up is higher in immigrants than native born Swedes. We then applied a path analysis approach to explore whether the relationship between immigrant status and chronic pain is mediated by mood status (i.e., anxiety and depression). The unique contribution of our study to the literature lies in the exploration of the causal pathway between immigration and chronic health condition outcomes, and in particular in investigating the role of mental health problems in the association between immigrant status and chronic pain.

## Methods

### Participants and procedures

SwePain, a large population-based study with a two-year follow-up, uses data from a sampling frame based on the Swedish Total Population Register (TPR). The sample frame consisted of 404,661 individuals who were 16–85 years old and living in south-eastern Sweden. The TPR uses a representative stratified random sample of 34,000 individuals of the sample frame [26–29]. The random sampling was stratified by sex and municipality to reach individuals living in urban and rural areas [29]. The analytical procedures of the sample design methods and survey questionnaire including items of the SwePain cohort have been described in detail elsewhere [26–29]. Data were collected by Statistics Sweden (SCB) [30]. The selected individuals received a postal questionnaire in March 2013, which could be returned either by post or electronically. The collection of questionnaires ended in May 2013. Follow-up data were collected 2 years later [26, 27]. The surveys at baseline and follow -up included the same questions.

The sample consisted of 15,563 individuals (46% men, 54% women). These individuals completed and returned the questionnaire at baseline for a response rate of 46% (Additional File 1: Supplementary Table 1). The response rate at baseline was lower among men, single people, and immigrants (Additional File 1: Supplementary Table 1) [26]. At the two-year follow-up, from 15,563 individuals who participated at baseline, 11386 individuals (55% women) completed and returned the questionnaire, a response rate of 73%. The response rate at follow-up was lower among men, single people, younger ages, secondary educated, immigrants, and individuals with depression and anxiety (Additional File 1: Supplementary Table 1) [26].

The study was approved by the local ethics committee of Linköping University, Sweden (Dnr: 2011 72/31). This study conformed to STROBE recommendations (Additional File 2).

### Measurements

#### Outcomes

The primary outcome of interest was the presence of chronic pain (CP) as defined using a single question based on the duration of pain [31]: ‘Do you frequently (usually) have pain lasting more than three months?’ (‘yes’ or ‘no’). Respondents were considered to have CP if they answered ‘yes’, and respondents were considered to have no chronic pain (NCP) if they answered ‘no’. This definition has the advantage that it is clear [31].

Secondary outcomes were the presence of chronic widespread pain (CWSP) and severe CP. The participants with pain marked the site of their pain during the previous 7 days on a body chart divided into 45 sections (22 on the front and 23 on the back) [29]. One marked area corresponds to 1 pain site; hence, the maximum number of pain sites was 45. Based on these 45 pain sites, 23 anatomical regions were determined and a total pain index, ranging from 0 to 23, was considered [26]. CWSP then, was defined as having CP in at least two anatomical regions in two contralateral limbs and the axial skeleton, which was equally marked on the front and the back of the manikin [26, 29]. We used a slightly modified definition of CWSP developed by MacFarlane and co-workers [32]. MacFarlane et al. [32] define widespread pain in limbs to be present ‘if there are at least two painful sections (in two contralateral limbs)’, a definition that does not require pain to be marked equally on the front and back of the body. Therefore, our study uses a more rigorous definition of widespread pain.

Severe CP was defined based on their pain intensity using a numeric rating scale (NRS) for the previous 7 days with anchors of 0 (no pain) and 10 (worst imaginable pain) [33]. Scores 0–3, 4–6, and 7–10 correspond to no/mild, moderate, and severe pain. We defined severe CP if the score was above 7 in the NRS. The NRS has provided good validity [34].

#### Exposures

In this study, we use the term ‘immigrant’ to denote individuals who were not born in Sweden (i.e., foreign-born or first-generation immigrants). We defined immigration exposure by immigration status according to information in the TPR and data based on country of birth from SCB [30]. Hence, respondents were classified as foreign-born if they were born outside of Sweden and Swedish-born if they were born in Sweden.

#### Covariates

In addition to baseline CP, CWSP, and severe CP, we examined seven baseline covariates as potential confounders: age, sex (women vs. men), marital status (married vs. other), education level (university vs. other), financial hardship (i.e., management of unforeseen financial adversity; yes vs. no), anxiety, and depression. These covariates were selected based on known associations between these factors and both CP and immigrants [12, 13, 26, 35]. Age, sex, marital status, and education level were recorded from the respondents’ answers in the postal survey. Financial hardship was measured by a single question: ‘If you should suddenly find yourself in an unforeseen situation where you had to acquire 14,000 SEK in 1 week, could you manage it? (yes, or no)’ [30]. This question is a measure of the financial situation by SCB and it can be considered an economic index showing financial hardship if the answer is no [30]. To evaluate anxiety and depression, we used the General Well-Being Scale (GWBS) [36]. The GWBS consists of 18 items with a total score ranging from 0 to 110 (high score indicating positive well-being and a low score indicating distress). The interval 0–60 reflects severe distress, 61–72 moderate distress, and 73–110 positive well-being. The first 14 questions use a six-point rating scale (ranging from 0 to 5) that represents intensity or frequency, and the remaining four items use an 11-point rating scale with the end-points 0 (very concerned) and 10 (not concerned at all) [37]. The instrument has provided good internal consistency, test-retest reliability, and validity [36]. GWBS can also produce six subscales [37]: Anxiety, Depression, Positive well-being, Self-control, Vitality, and General health. In this study, we used the subscales Anxiety and Depression.

## Data analysis

All statistical analyses were performed using IBM SPSS Statistics (version 25.0; IBM Inc., New York, USA) and R statistical language and environment (version 3.6.1) using the lavaan package [38]. Two-sided statistical tests were used and a *P* < 0.05 was considered significant. We calculated means and standard deviations (SDs) for continuous variables and frequencies with percentages (n; %) for categorical variables.

To examine the predictive association between baseline immigration status (foreign-born vs Swedish-born) with the pain outcomes at follow-up (presence of CP, CWSP, and severe CP), we used logistic regression models under the Generalized Estimated Equations (GEE) with robust standard errors and a logit link function, while we employed an unstructured correlation matrix [39]. GEE is a flexible method for longitudinal analysis and can be used to analyse correlated data with binary, discrete, or continuous outcomes, also considering the dependency between repeated measures. This technique also allows all participants to be included in the analysis even when data are missing [39]. Particularly, it allows missing values within a subject without losing all the data from the subject, and time-varying predictors that can appear in the model [40]. The statistical significance of the models was determined using the Wald test [41]. For this analysis, we adjusted for unequal possibilities of sample selection by weighting cases regarding age, strata, gender, and city. These weights were calculated by SCB [30]. We produced two models per outcome of interest and per immigration status: one unadjusted in which crude ORs with corresponding 95% (CIs) were calculated; and one adjusted model including time independent variable of sex (women vs. men), and time dependent variables of age, marital status (married vs. other), financial hardship (yes vs. no), anxiety, depression, and changes in CP, CWSP, and severe CP. We also performed a sensitivity analysis excluding those with missing information on the baseline variable of the financial hardship and one including only those who had developed chronic pain at follow-up.

We then explored whether the relationship between immigrant status and chronic pain is mediated by mood status (i.e., anxiety and depression) via a path analysis approach. Path analysis can be used to describe the directed dependencies among a set of variables and can estimate both the magnitude and significance of causal links between variables [42]. For this analysis we used baseline data for immigration status and covariates while we used the follow-up data for the outcomes of interest i.e., all three chronic pain conditions. Participants with missing values were excluded from this analysis. The final sample size for the analysis after excluding missing values was *n* = 11,152 for CP and CWSP while the final sample size for severe CP was *n* = 6870. Path models identification (i.e., just-identified model, over-identified model, and under-identified model) were based on degrees of freedom (df) which are related to the number of parameter estimates. The models df must be equal or bigger than 0 [43]. We tested the path model using the maximum likelihood estimation using the fit indices proposed by Hu and Bentler [44] as well as Barrett [45]. Briefly, we used the Chi-Square (χ^2^) value, which is the traditional measure for evaluating overall model fit and ‘assesses the magnitude of discrepancy between the sample and fitted covariances matrices’ [44]. A good model fit should provide an insignificant result at a 0.05 threshold [45]. Other indicators were the Tucker Lewis Index (TLI), the normed fit index (NFI), the non-normed fit index (NNFI), the comparative fit index (CFI), and the goodness-of-fit index (GFI), which shows the model fit relative to the null model. Typically, all indices are considered acceptable when estimates ≥0.90 [44]. The root mean square error of approximation (RMSEA), and the standardized root mean square residual (SRMSR) were also included. For both latter indices, estimates ≤0.05 were considered a good fit. We presented three models: one for CP, one for CWSP, and one for severe CP. We tested the mediation effect of mood status (i.e., anxiety and depression) with bootstrapping procedures using the mediate function from the mediation package in R [46]. We also transformed the standardized regression coefficients (beta) into ORs.

## Results

### Population characteristics

The total sample consisted of 8412 women (54%) and 7151 men (46%) and the mean age was 51.6 (SD = 18.4) years (Table 1). Of those, 1470 (10%) were foreign-born (i.e., immigrants) and 14,093 (90%) were Sweden-born.

**Table 1:** Baseline and 2 years follow-up characteristics by immigration status: Swedish-born population and Foreign-born population

Characteristics, n (%) otherwise stated	Swedish-born population (*N* = 14,093; 90%)	Foreign-born population (*N* = 1470; 10%)	***p value***
**Baseline**			
Age (mean, SD)	51.9 (18.5)	48.9 (17.2)	< 0.001
Women	7599 (54)	813 (55)	0.32
Married	7015 (50)	810 (55)	0.95
University education	5062 (37)	527 (38)	0.66
Financial hardship	1512 (11)	451 (32)	< 0.001
Pain intensity (mean, SD)	5.2 (1.9)	5.4 (2.1)	0.21
GWBS Anxiety (mean, SD)	7.1 (5.1)	8.9 (5.3)	< 0.001
GWBS Depression (mean SD)	4.9 (3.7)	6.5 (4.3)	< 0.001
CP	5285 (38)	632 (44)	0.007
CWSP	1012 (7)	174 (12)	< 0.001
Severe CP	391 (27)	54 (34)	0.06
**2 years follow-up**	**Swedish-born population (*N*** **= 10,496; 92%)**	**Foreign-born population (*N*** **= 890; 8%)**	
Age (mean, SD)	56.0 (17.6)	53.3 (16.7)	< 0.001
Women	5674 (55)	483 (56)	0.90
Married	5601 (53)	504 (56)	< 0.001
University education	4058 (39)	356 (42)	0.43
Financial hardship	846 (8)	213 (25)	< 0.001
Pain intensity (mean, SD)	4.7 (1.9)	5.3 (2.1)	< 0.001
GWBS Anxiety (mean, SD)	6.9 (5.1)	8.4 (5.4)	< 0.001
GWBS Depression (mean SD)	4.9 (3.7)	6.1 (4.3)	< 0.001
CP	4083 (39)	395 (45)	< 0.001
CWSP	793 (8)	101 (11)	< 0.001
Severe CP	951 (20)	158 (32)	< 0.001

Notes: CP=Chronic pain, CWSP=Chronic widespread pain, CGWBS = General Well-Being Scale, SD = standard deviation

In the sample of 1470 immigrants, 72 (5%) were from Africa, 449 (30%) from Asia and Oceania, 586 (40%) from Europe, 258 (17%) from other Nordic countries, 35 (3%) from North America, and 70 (5%) were from South America.

Foreign-born were younger (*P* < 0.001) with a higher level of anxiety as well as depression (both *P* < 0.001) compared to Swedish-born at both baseline and follow-up. Further baseline and follow-up characteristics of the sample are presented in Table 1 and in Additional File 1. Pain intensity, anxiety, and depression were fairly stable over time for both foreign-born and Swedish-born cohorts (Table 1).

In the total sample, 4478 individuals (40%) had CP at follow-up, including 894 individuals (8%) with CWSP and 1109 (21%) with severe CP. The prevalence of CP, CWSP and severe CP at follow-up was significantly higher in foreign-born compared to Swedish-born (45, 11, and 32% vs 39, 8, and 20% respectively). Baseline and follow-up percentages of CP, CWSP, and severe CP for both foreign-born and Swedish-born cohorts are presented in Table 1.

### Predictive analysis

The results of the GEE analyses (unadjusted and adjusted) are presented in Table 2. Both the unadjusted and adjusted models showed that immigrants had an elevated risk for all chronic pain outcomes, even though the ORs were attenuated after adjustments. For example, the adjusted OR for CP was 1.18 (95%:1.04–1.33), the adjusted OR for CWSP was 1.39 (95% CI:1.15–1.69) and the adjusted OR for severe CP was 1.51 (95%:1.23–1.87) (Table 2). The results from the sensitivity analysis showed similar patterns with the main analysis (data not shown).

**Table 2:** Unadjusted and Adjusted odds ratios for CP, CWSP, and severe CP after 2 years by immigration status: Swedish-born population and Foreign-born population

			Unadjusted			Adjusted		
	N	%	OR	95% CI	*p* value	OR	95%CI	*p* value
**CP**								
Swedish-born	4083	39	1 [reference]			1 [reference]		
Foreign-born	395	45	1.26	1.14–1.39	< 0.001	1.18	1.04–1.33	0.009
**CWSP**								
Swedish-born	793	8	1 [reference]			1 [reference]		
Foreign-born	101	11	1.66	1.42–1.94	< 0.001	1.39	1.15–1.69	0.001
**Severe CP**								
Swedish-born	951	20	1 [reference]			1 [reference]		
Foreign-born	158	32	1.76	1.47–2.09	< 0.001	1.51	1.23–1.87	< 0.001

Notes: CP=Chronic pain, CWSP=Chronic widespread pain, OR = Odds ratio, CI=Confidence interval, Adjusted = adjusted for age, sex, marital status, education, financial hardship, anxiety, depression, and changes in CP, CWSP, and severe CP

### Path analysis

**Fig. 1:**
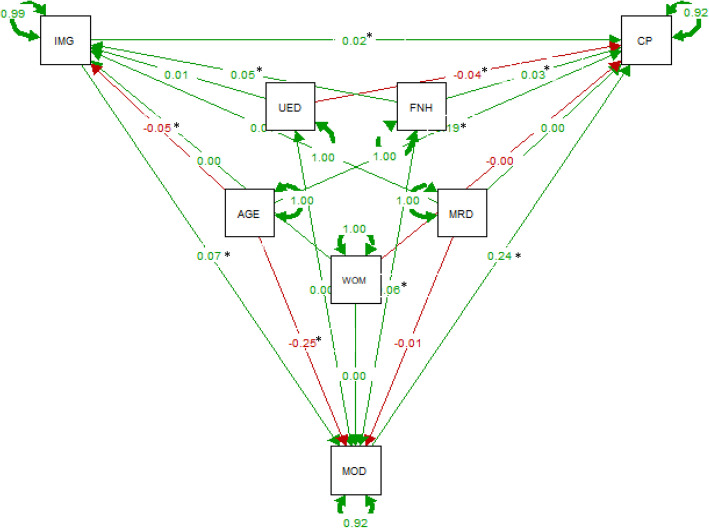
Path analysis results on the effect of the relationship between baseline immigrant status and chronic pain at follow up with baseline mood i.e., anxiety and depression as mediators. (Standardized estimates, *N* = 11,152) Notes: AGE = age, CP = chronic pain, IMG = Immigrants, UED = university education, WOM = women, MRD = married, MOD = mood (anxiety and depression), FNH = financial hardship. * significant at *P* < .05

**Fig. 2:**
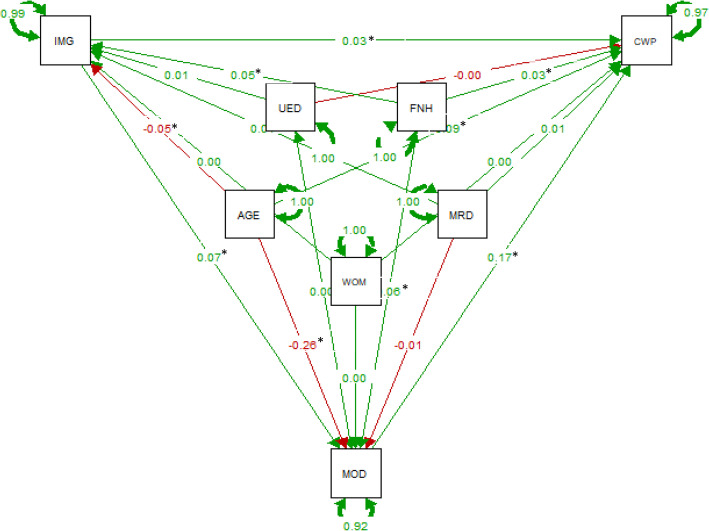
Path analysis results on the effect of the relationship between baseline immigrant status and chronic widespread pain at follow-up with baseline mood i.e., anxiety and depression as mediators. (Standardized estimates, N = 11,152) Notes: AGE = age, CWP = chronic pain, IMG = Immigrants, UED = university education, WOM = women, MRD = married, MOD = mood (anxiety and depression), FNH = financial hardship. * significant at *P* < .05

**Fig. 3:**
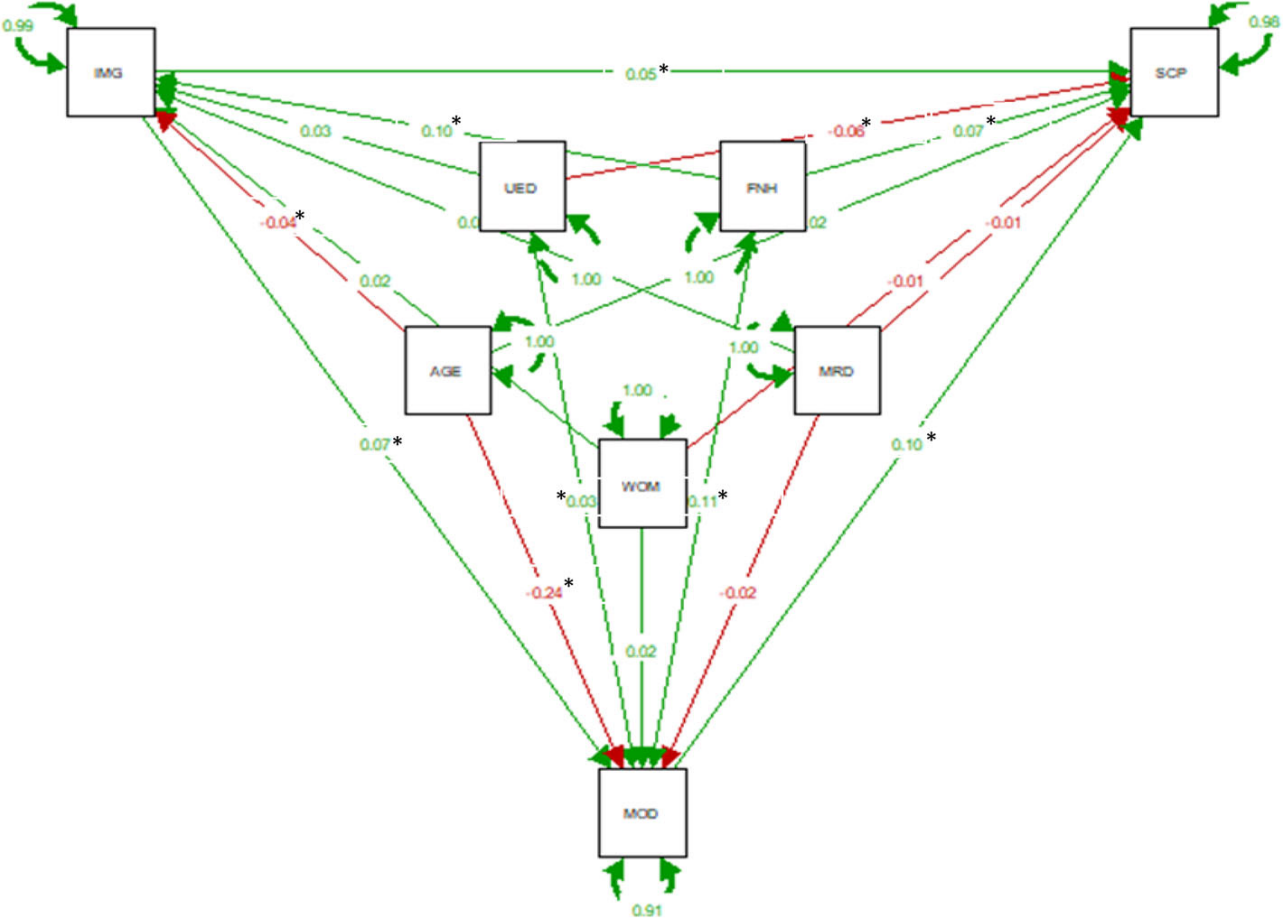
Path analysis results on the effect of the relationship between baseline immigrant status and severe chronic pain at follow up with baseline mood i.e., anxiety and depression as mediators. (Standardized estimates, *N* = 6870) Notes: AGE = age, SCP = severe chronic pain, IMG = Immigrants, UED = university education, WOM = women, MRD = married, MOD = mood (anxiety and depression), FNH = financial hardship. * significant at *P* < .05

The overall findings from path analysis are presented in Additional File 1: Supplementary Tables 2, 3 and 4, and the standardized regression coefficients (bstd) are illustrated in Figs. 1, 2 and 3.

All models had a very good fit with a non-significant Chi-square (CP:[χ2 (1)] = 1.854, *p* = 0.35; CWSP:[χ2 (1)] = 1.606, *p* = 0.44; and severe CP: [χ2 (1)] = 2.969, *p* = 0.08), indicating that the assumed path models are adequate for the data (i.e., the model and the data are not statistically significantly different). Based on the degrees of freedom all models were slightly overidentified allowing for the parameters to be estimated [43]. All output of the models indicated a very good fit to the data and all indices were within acceptable limits (Additional File 1: Supplementary Tables 2, 3 and 4).

#### Effects on chronic pain, chronic widespread pain, and severe chronic pain

Baseline age, immigration status, financial hardship, and mood variables had a direct significant increased effect on CP at follow-up (bstd =0.19, *p* < 0.001; bstd =0.02, *p* = 0.01; bstd =0.03, *p* < 0.001; bstd =0.02, *p* = 0.01 respectively), while baseline university education had a direct decreased effect on CP at follow-up (bstd = − 0.04, *p* < 0.001; Fig. 1 and Additional File 1: Supplementary Table 2). Almost identical correlation patterns were observed for CWSP and severe CP at follow-up (Figs. 2 and 3 and Additional File 1: Supplementary Tables 3 and 4).

#### Effects on mood status

Baseline immigration status and financial hardship had a direct significant increased effect on baseline mood, while baseline age had a direct decreased effect on baseline mood in all three models per pain outcome of interest (Figs. 1, 2 and 3, and Additional File 1: Supplementary Tables 2–4). For the model accounted for severe CP at follow-up, baseline university education had also a direct increased effect on baseline mood (bstd =0.03, *p* = 0.04, Fig. 3 and Additional File 1: Supplementary Table 4).

#### Effects on immigration status

Baseline financial hardship had a direct significant increased effect on baseline immigration status, while baseline age had a direct significant decreased effect on baseline immigration status in all three models per pain outcome of interest (Figs. 1, 2 and 3, and Additional File 1: Supplementary Tables 2–4).

#### Mediation analysis

**Fig. 4:**
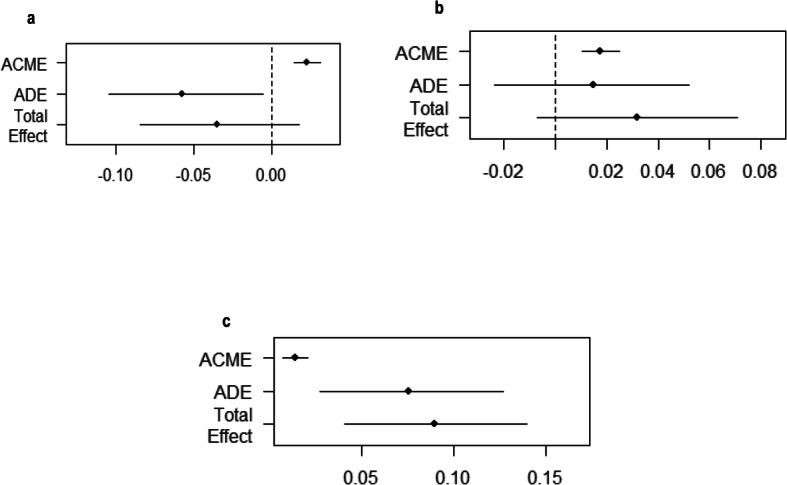
Plots of casual mediation analysis of mood for chronic pain (**a**), chronic widespread pain (**b**) and severe chronic pain (**c**). Notes: ACME = Average Causal Mediation Effect, or the indirect effect, ADE = Average Direct Effect, or the direct effect, Total Effect = the total effect. The upper and lower confidence intervals based upon the quantiles of the bootstrapped distribution

Finally, baseline mood i.e., anxiety and depression, as shown Figs. 1, 2 and 3, mediated the relationship between baseline immigrant status and all chronic pain outcomes at follow-up. We tested the significance of this effect using bootstrapping procedures [46]. For CP, as Fig. 4a illustrates the effect of baseline immigration status on chronic pain at follow-up was inconsistently mediated via mood. The bootstrapped average causal mediation effects (ACME) was 0.02 (*p* < 0.001), and the average direct effects (ADE) was − 0.06 (*p* = 0.02). Thus, both direct and indirect effect were statistically significant, but not in the same direction. However, the total effect was not statistically significant (*p* = 0.17). For CWSP the effect of baseline immigration status on chronic pain at follow-up was fully mediated via mood. The ACME was 0.02 (*p* < 0.001), indicating a significant indirect effect, but both ADE and total effect were not statistically significant (both *p* > 0.05, Fig. 4b). For severe CP, as Fig. 4c illustrates the effect of baseline immigration status on severe CP at follow-up was partly mediated via mood. The ACME, ADE, and total effect were statistically significant (all *p* < 0.001).

## Discussion

Based on our large cohort of the general population, a high prevalence of chronic pain, chronic widespread pain, and severe chronic pain was observed among first generation immigrants 2 years after baseline data collection. Moreover, an increased risk of having any chronic pain outcome was found. Both unadjusted and adjusted models and sensitivity analysis showed similar results. The adjusted risk was almost one and a half times higher for chronic widespread pain and severe chronic pain for migrants compared to Swedes. Our exploratory analysis also found that baseline financial hardship, depression, and anxiety may play an important role in chronic pain among immigrants. Especially, baseline mood aspects seem to mediate the relationships between baseline immigration status and chronic pain outcomes at follow-up. Mood status fully mediates the relationship between immigration and chronic widespread pain, while in the case of chronic pain it may also have a suppressor effect [47].

To our knowledge, this large population-based study is the first study evaluating three common chronic pain outcomes to spotlight the role of immigration in chronic health conditions such as chronic pain by comparing the chronic pain ORs in immigrants with native populations. In addition, our study is unique in the sense that it sheds light on the pathway between immigration and chronic health outcomes, exploring the mediating role of mental health problems in the association between chronic pain and immigrant status. Our results now provide important evidence in an otherwise sparse area of study.

Overall, the present study confirmed the findings regarding inequalities in chronic pain prevalence among immigrant populations. Similar to our study, Kurita et al. [19] documented a higher prevalence of pain in individuals with a foreign background compared to native Danes. This was also the case for the studies conducted by Soares et al. [18] and of Choudhury et al. [20]. The latter study found that chronic widespread pain was more common and more severe in the Bangladeshi than in the white population in East London. Our study shows that being an immigrant is not only associated with increased chronic pain, but also other relevant factors (i.e., age and financial hardship) and more importantly mental health conditions such as anxiety and depression may play an important role in the experience of chronic pain among immigrants. Moreover, our results are in agreement with previous studies that suggest immigrants have higher odds of chronic musculoskeletal pain [19–21]. However, the majority of the earlier studies used a cross-sectional study design, making a direct comparison with our findings not fully relevant. Our results are partly in agreement with a recent population-based study in Germany, showing that although ‘migration background’ as per official statistics definition is not related with increased mental health problems, identification as an immigrant (self and/or by others) was found as significant predictor for PTSD and depression [48]. A previous report investigating health-related quality of life outcomes have also documented important disparities between racial/ethnic groups related to the experience and management of pain [23]. Taking into consideration our findings, our study did not seem to follow the hypothesis of a ‘healthy immigrant effect’ [24]. Today, there is extensive ongoing research about the above mentioned phenomenon, yet the findings have been inconclusive [25, 49–51].

Our path analysis showed that immigration status, along with age, financial hardship, and mood variables were associated with a higher risk for all chronic pain outcomes, whilst on the other hand, university education was associated with a lower risk for chronic pain. These findings seem to be in concordance with previous literature from Sweden which showed that experiencing pain was more severe in the older immigrants, suffering from depression with a background of limited education [52]. The path analysis in our study also highlighted the importance of co-existing mental health problems in the experience of chronic pain, since the associations between immigrant status and all three pain outcomes were mediated by mental health status, i.e., anxiety and depression. Anxiety and depression are known to be more prevalent in immigrant populations and have been associated with increased pain [52]. In general, our results agree with earlier findings showing strong associations between anxiety, depression, and socio-economic situation and future chronic pain [11, 26, 53, 54]. Other well-known sociodemographic factors related to future chronic pain outcomes such as age, sex, and education [26, 35, 53, 54] were also confirmed in our analysis. Future research should thoroughly investigate immigration-related factors including a wide-range of sociodemographic and health-related factors that may contribute to the health status among immigrants.

The results of this study should be interpreted taking into consideration some limitations. While our study used a longitudinal study design in conjunction with large and representative sample size, the response rate was low and the proportion of the immigrant population was relatively very low compared to the native population (10% vs 90%, respectively). This low proportion alongside the declined response rate at follow-up among immigrants in our data may underestimate the observed predictive associations between immigrant status and chronic pain outcomes. Likewise, there was great heterogeneity among immigrants. Thus, our findings should be interpreted with caution also considering that the short follow-up (i.e., 2 years) may not be long enough to properly explore the changes of chronic pain status. Moreover, as this study collected data using postal surveys rather than interviews, it was not possible to include refugees or to examine other immigration-related factors such as language skills, age at immigration, second immigrant generation, and acculturation status, factors that have been proven to affect the relationship between immigration and health status [4, 7, 17]. Furthermore, the postal design of the study means that some of the most mentally unwell immigrants (who in general have more frequent and severe mental health difficulties seemingly associated with higher levels of chronic pain) may have found it more difficult to complete the questionnaires and therefore immigrants with severe chronic widespread pain/chronic severe pain might have been under-represented in the survey response and outcomes. Finally, the hypothesized relationships between the variables may be in different directions. For example, we found that mood (anxiety and depression) mediates the relationship between immigrant status and pain; yet it could also be plausible that pain would partially explain the relationship between immigrant status and mood (anxiety and depression). Generally, mediation is ideal in the context of experimental designs (which have many controls); accordingly, it should be fully acknowledged that our study design/model was unable to account for many other potential explanations.

In conclusion, our study highlights the importance of evaluating the chronic pain prevalence and experience among immigrants and verifies a predictive association between immigrant status and increased risk of chronic pain, widespread pain, and severe chronic pain after adjustments for known risk factors. More importantly, our study provides health care practitioners with a deeper knowledge of the factors influencing the relationship between immigration status and chronic pain, which, in turn, could help enable targeted interventions better tailored to socio-economic and psychological status of immigrants with chronic pain. These findings are important because pain, anxiety, depression, and social factors like financial strain may lead to greater ill-health. Future research with larger samples should thoroughly investigate immigration-related factors including a wide-range of sociodemographic and health-related factors that may contribute to the health status among immigrants are needed to evaluate our findings, considering the difficulty of trans-cultural care.

## Supplementary information

**Additional file 1: Supplementary Table 1.** Description of the sociodemographic characteristics and study measures at both baseline and at the two-year follow-up and characteristics of non-participants at baseline and follow up. **Supplementary Table 2**. Path model’s parameters for chronic pain ^(1)^. **Supplementary Table 3.** Path model’s parameters for chronic widespread pain ^(1)^. **Supplementary Table 4.** Path model’s parameters for severe chronic pain ^(1)^.

**Additional file 2.** STROBE_checklist.

## Data Availability

Due to the Swedish law regarding the type of data including grounds of confidentiality and anonymity data cannot be available.

## Abbreviations

CI: Confidence Interval
CP: Chronic Pain
CWSP: Chronic Widespread Pain
GEE: Generalized Estimating Equation
GWBS: the General Well-Being Scale
NRS: Numeric Rating Scale
OR: Odds Ratio
SCB: Statistics Sweden (Swedish: Statistiska centralbyrån)
SD: Standard Deviation
STROBE: Strengthening the Reporting of Observational Studies in Epidemiology
TPR: Swedish Total Population Register
