# Supplementary material for: Predictive association between immigration status and chronic pain in the general population: results from the SwePain cohort
Source: BMC Public Health. 2020 Sep 29;20:1462. doi: 10.1186/s12889-020-09546-z (PMC7526181; doi:10.1186/s12889-020-09546-z)
Supplement: Supplementary file 1 — Additional file 1: Supplementary Table 1. Description of the sociodemographic characteristics and study measures at both baseline and at the two-year follow-up and characteristics of non-participants at baseline and follow up. Supplementary Table 2. Path model’s parameters for chronic pain (1). Supplementary Table 3. Path model’s parameters for chronic widespread pain (1). Supplementary Table 4. Path model’s parameters for severe chronic pain (1). [file 12889_2020_9546_MOESM1_ESM.docx]

**Supplementary Table 1.** Description of the sociodemographic characteristics and study measures at both baseline and at the two-year follow-up and characteristics of non-participants at baseline and follow up.

| **Characteristic**; n (%),  unless otherwise stated | **Number of answers on specific items** | **Participants at baseline (N=15563)** | **Number of answers on specific items** | **Participants**  **at follow -up**  **(baseline responses; N=11386)** | **Number of answers on specific items** | **Participants**  **at follow -up**  **(follow-up responses.**  **N =11386)** | **Non-Participants at baseline**  **(N=18437)** | **Non-Participants at follow -up**  **(N=4177)** |
| --- | --- | --- | --- | --- | --- | --- | --- | --- |
| **Age, years;** mean (SD) | 15563 | 51.6 (18.5) | 11386 | 53.8 (17.5) | 11384 | 55.8 (17.5) | - | 45.6 (19.5) |
| **Sex** | 15563 |  | 11386 |  | 11386 |  |  |  |
| Men |  | 7151 (46.0) |  | 5125 (45.0) |  | 5125 (45.0) | 9837 (54.0) | 2026 (48.5) |
| Women |  | 8412 (54.0) |  | 6261 (55.0) |  | 6261 (55.0) | 8382 (46.0) | 2151 (51.5) |
| **Civil status** | 15555 |  | 11381 |  | 11386 |  |  |  |
| Single |  | 5134 (33.0) |  | 3283 (28.8) |  | 3179 (27.9) | 9440 (51.8) | 1851 (44.3) |
| Married |  | 7825 (50.3) |  | 6104 (53.7) |  | 6105 (53.6) | 6347 (34.8) | 1721 (41.2) |
| Divorced |  | 1762 (11.3) |  | 1351 (11.9) |  | 1387 (12.2) | 1802 (9.9) | 411 (9.8) |
| Widowed |  | 834 (5.4) |  | 643 (5.6) |  | 715 (6.3) | 630 (3.5) | 191 (4.6) |
| **Educational level** | 15256 |  | 11205 |  | 11162 |  |  |  |
| Elementary school |  | 3442 (22.6) |  | 2571 (22.9) |  | 2491 (22.3) | - | 871 (21.5) |
| Secondary school or vocational training |  | 6225 (40.8) |  | 4327 (38.6) |  | 4257 (38.2) | - | 1898 (46.9) |
| College or university |  | 5589 (36.6) |  | 4307 (38.5) |  | 4414 (39.5) | - | 1282 (31.6) |
| **Financial hardship** | 15115 |  | 11252 |  | 11260 |  |  |  |
| Yes |  | 1963 (12.8) |  | 1206 (10.7) |  | 1059 (9.4) | - | 757 (18.5) |
| No |  | 13383 (87.2) |  | 10046 (89.3) |  | 10201 (90.6) | - | 3337(81.5) |
| **Immigration status** | 15346 |  | 11386 |  | 11386 |  |  |  |
| Swedish-born |  | 14093 (90.6) |  | 10496 (92.2) |  | 10496 (92.2) | 14475 (79.5) | 3597 (86.1) |
| Foreign-born |  | 1470 (9.4) |  | 890 (7.8) |  | 890 (7.8) | 3744 (20.5) | 580 (13.9) |
| **Pain intensity; (mean, SD)** | 6870 | 4.8 (2.0) | 4993 | 4.7 (1.9) | 5305 | 4.7 (1.9) |  | 4.9 (2.1) |
| **GWBS Anxiety (mean, SD)** | 14969 | 7.3 (5.1) | 11012 | 6.9 (5.1) | 10935 | 7.0 (5.1) | - | 8.2 (5.3) |
| **GWBS Depression (mean SD)** | 15020 | 5.1 (3.8) | 11060 | 4.9 (3.6) | 10959 | 5.0 (3.7) | - | 5.6 (4.1) |
| **CP** | 15251 | 5917 (92.4) | 11165 | 4318 (38.7) | 11152 | 4478 (40.2) | - | 1599 (39.1) |
| **CWSP** | 15251 | 1186 (7.6) | 11165 | 861 (7.6) | 11152 | 894 (7.9) | - | 325 (7.8) |
| **Severe CP** | 6870 | 1437 (20.9) | 4993 | 1009 (20.2) | 5305 | 1109 (20.9) | - | 428 (22.8) |

Notes: CP=Chronic pain, CWSP=Chronic widespread pain, CGWBS= General Well-Being Scale, SD=standard deviation.

| **Supplementary Table 2**. Path model's parameters for chronic pain ^(1)^ | | | | | | | |
| --- | --- | --- | --- | --- | --- | --- | --- |
|  | |  | **95% C.I.** | |  |  |  |
|  | | **Beta^(2)^** | **Lower** | **Upper** | **z-value** | **p** | **OR ^(3)^** |
| CP at follow up~ | |  |  |  |  |  |  |
| Baseline factors | MOD | 0.24 | 0.22 | 0.25 | 33.301 | <0.001 | 1.27 |
|  | IMG | 0.02 | 0.00 | 0.03 | 2.493 | 0.01 | 1.02 |
|  | FNH | 0.03 | 0.01 | 0.04 | 3.592 | <0.001 | 1.03 |
|  | MRD | 0.00 | -0.01 | 0.01 | 0.037 | 0.97 | 1.00 |
|  | WOM | -0.00 | -0.01 | 0.01 | -0.337 | 0.73 | 1.00 |
|  | AGE | 0.19 | 0.17 | 0.20 | 24.805 | <0.001 | 1.21 |
|  | UED | -0.04 | -0.05 | -0.02 | -4.888 | <0.001 | 0.96 |
| MOD at baseline ~ | |  |  |  |  |  |  |
| Baseline factors | IMG | 0.07 | 0.05 | 0.08 | 8.937 | <0.001 | 1.07 |
|  | FNH | 0.06 | 0.04 | 0.07 | 7.794 | <0.001 | 1.06 |
|  | MRD | -0.01 | -0.02 | 0.00 | -1.558 | 0.11 | 0.98 |
|  | WOM | 0.00 | -0.01 | 0.01 | 0.422 | 0.67 | 1.00 |
|  | AGE | -0.25 | -0.27 | -0.24 | -33.553 | <0.001 | 0.97 |
|  | UED | 0.00 | -0.01 | 0.01 | 0.282 | 0.77 | 1.00 |
| IMG at baseline~ | |  |  |  |  |  |  |
| Baseline factors | FNH | 0.05 | 0.36 | 0.06 | 5.859 | <0.001 | 1.05 |
|  | MRD | 0.00 | -0.01 | 0.02 | 0.913 | 0.36 | 1.01 |
|  | WOM | 0.00 | -0.01 | 0.02 | 0.427 | 0.66 | 1.00 |
|  | AGE | -0.05 | -0.06 | -0.03 | -5.832 | <0.001 | 0.95 |
|  | EDU | 0.01 | -0.01 | 0.02 | 0.912 | 0.36 | 1.01 |
| 1. χ2 (18) =3047.136, p < 0.001, TLI = 0.999, NFI =0.999, NNFI =0.999, CFI = 0.999, GFI = 0.999, SRMR =0.001, RMSEA =0.000   Notes:AGE=age, CP= Chronic pain, CI=Confidence interval, IMG=Immigrants, UED= university education, WOM= women, MRD=married, MOD= mood (anxiety and depression), FNH=financial hardship, OR=Odds ratio, | | | | | | | |
| 1. Completely standardized solution beta coefficients (estimates of parameters if the variances are unity). | | | | | | | |
| (3) Exponential form of beta. | | | | | | | |

| **Supplementary Table 3.** Path model's parameters for chronic widespread pain ^(1)^ | | | | | | | |
| --- | --- | --- | --- | --- | --- | --- | --- |
|  | |  | **95% C.I.** | |  |  |  |
|  | | **Beta^(2)^** | **Lower** | **Upper** | **z-value** | **p** | **OR ^(3)^** |
| CWP at follow up~ | |  |  |  |  |  |  |
| Baseline factors | MOD | 0.17 | 0.15 | 0.18 | 20.493 | <0.001 | 1.18 |
|  | IMG | 0.03 | 0.01 | 0.04 | 3.247 | 0.001 | 1.03 |
|  | FNH | 0.03 | 0.01 | 0.04 | 3.153 | 0.002 | 1.03 |
|  | MRD | 0.01 | -0.00 | 0.02 | 1.733 | 0.08 | 1.01 |
|  | WOM | 0.00 | -0.01 | 0.02 | 0.507 | 0.61 | 1.00 |
|  | AGE | 0.09 | 0.07 | 0.10 | 12.066 | <0.001 | 1.09 |
|  | UED | -0.00 | -0.02 | 0.01 | -0.321 | 0.75 | 0.99 |
| MOD at baseline ~ | |  |  |  |  |  |  |
| Baseline factors | IMG | 0.07 | 0.05 | 0.09 | 8.881 | <0.001 | 1.07 |
|  | FNH | 0.06 | 0.05 | 0.08 | 8.056 | <0.001 | 1.06 |
|  | MRD | -0.01 | -0.02 | 0.00 | -1.514 | 0.13 | 0.99 |
|  | WOM | 0.00 | -0.01 | 0.02 | 0.367 | 0.71 | 1.00 |
|  | AGE | -0.26 | -0.27 | -0.24 | -33.863 | <0.001 | 0.77 |
|  | UED | 0.00 | -0.01 | 0.02 | 0.627 | 0.53 | 1.00 |
| IMG at baseline~ | |  |  |  |  |  |  |
| Baseline factors | FNH | 0.05 | 0.04 | 0.07 | 6.206 | <0.001 | 1.05 |
|  | MRD | 0.01 | -0.01 | 0.02 | 0.709 | 0.48 | 1.01 |
|  | WOM | 0.01 | -0.01 | 0.02 | 0.669 | 0.50 | 1.01 |
|  | AGE | -0.05 | -0.07 | -0.03 | -6.183 | <0.001 | 0.95 |
|  | EDU | 0.01 | -0.01 | 0.02 | 0.766 | 0.44 | 1.01 |
| 1. χ2 (18) = 2221.684, p < 0.001, TLI = 0.999, NFI =0.999, NNFI =0.999, CFI = 0.999, GFI = 0.999, SRMR =0.001, RMSEA =0.000 Notes:AGE=age, CI=Confidence interval, CWP= chronic widespread pain, IMG=Immigrants, UED= university education, WOM= women, MRD=married, MOD= mood (anxiety and depression), FNH=financial hardship, OR=Odds ratio. | | | | | | | |
| 1. Completely standardized solution beta coefficients (estimates of parameters if the variances are unity). | | | | | | | |
| (3) Exponential form of beta. | | | | | | | |

| **Supplementary Table 4.** Path model's parameters for severe chronic pain ^(1)^ | | | | | | | | |
| --- | --- | --- | --- | --- | --- | --- | --- | --- |
|  | |  | **95% C.I.** | |  |  |  |  |
|  | | **Beta^(2)^** | **Lower** | **Upper** | **z-value** | **p** | **OR ^(3)^** |  |
| SCP at follow up~ | |  |  |  |  |  |  |  |
| Baseline factors | MOD | 0.10 | 0.07 | 0.13 | 6.399 | <0.001 | 1.11 |  |
|  | IMG | 0.05 | 0.03 | 0.09 | 3.614 | <0.001 | 1.05 |  |
|  | FNH | 0.07 | 0.04 | 0.10 | 4.597 | <0.001 | 1.07 |  |
|  | MRD | -0.01 | -0.03 | 0.02 | -0.429 | 0.67 | 0.99 |  |
|  | WOM | -0.01 | -0.04 | 0.01 | -0.911 | 0.36 | 0.99 |  |
|  | AGE | 0.02 | -0.01 | 0.05 | 1.289 | 0.20 | 1.02 |  |
|  | UED | -0.06 | -0.09 | -0.03 | -3.736 | <0.001 | 0.94 |  |
| MOD at baseline ~ | |  |  |  |  |  |  |  |
| Baseline factors | IMG | 0.07 | 0.04 | 0.10 | 4.798 | <0.001 | 1.07 |  |
|  | FNH | 0.11 | 0.08 | 0.14 | 7.640 | <0.001 | 1.12 |  |
|  | MRD | -0.02 | -0.05 | 0.00 | -1.819 | 0.07 | 0.98 |  |
|  | WOM | 0.01 | -0.01 | 0.02 | 1.362 | 0.17 | 1.01 |  |
|  | AGE | -0.24 | -0.27 | -0.21 | -16.415 | <0.001 | 0.78 |  |
|  | UED | 0.03 | 0.01 | 0.06 | 2.103 | 0.04 | 1.03 |  |
| IMG at baseline~ | |  |  |  |  |  |  |  |
| Baseline factors | FNH | 0.10 | 0.06 | 0.13 | 5.571 | <0.001 | 1.11 |  |
|  | MRD | 0.02 | -0.01 | 0.05 | 1.375 | 0.17 | 1.02 |  |
|  | WOM | 0.02 | -0.01 | 0.05 | 1.733 | 0.08 | 1.02 |  |
|  | AGE | -0.04 | -0.07 | -0.01 | -2.616 | 0.009 | 0.96 |  |
|  | EDU | 0.03 | -0.01 | 0.06 | 1.730 | 0.08 | 1.03 |  |
| 1. χ2 (18) = 665.608, p < 0.001, TLI = 0.945, NFI =0.996, NNFI =0.945, CFI = 0.997, GFI = 0.999, SRMR =0.004, RMSEA =0.020 Notes:AGE=age, CI=Confidence interval, CWP= chronic widespread pain, IMG=Immigrants, UED= university education, WOM= women, MRD=married, MOD= mood (anxiety and depression), FNH=financial hardship, OR=Odds ratio. | | | | | | | | |
| 1. Completely standardized solution beta coefficients (estimates of parameters if the variances are unity). | | | | | | | | |
| (3) Exponential form of beta. | | | | | | | | |
